# Why people keep watching: neurophysiologic immersion during video consumption increases viewing time and influences behavior

**DOI:** 10.3389/fnbeh.2022.1053053

**Published:** 2022-12-13

**Authors:** Li-Hsin Lin, Rainita Narender, Paul J. Zak

**Affiliations:** ^1^Center for Neuroeconomics Studies, Claremont Graduate University, Claremont, CA, United States; ^2^Department of Economics, McMaster University, Hamilton, ON, Canada

**Keywords:** persuasion, immersion, free choice, charity, prediction

## Abstract

Streaming services provide people with a seemingly infinite set of entertainment choices. This large set of options makes the decision to view alternative content or stop consuming content altogether compelling. Yet, nearly all experimental studies of the attributes of video content and their ability to influence behavior require that participants view stimuli in their entirety. The present study measured neurophysiologic responses while participants viewed videos with the option to stop viewing without penalty in order to identify signals that capture the neural value of content. A post-video behavioral choice was included to reduce the likelihood that measured neurophysiologic responses were noise rather than signal. We found that a measure derived from neurophysiologic Immersion predicted how long participants would watch a video. Further, the time spent watching a video increased the likelihood that it influenced behavior. The analysis indicates that the neurologic value one receives helps explain why people continue to watch videos and why they are influenced by them.

## Introduction

Audiences want to be immersed in content, from movies to TV, to music (Bernhaupt, 2010; Johnson et al., 2016). Streaming services such as YouTube, Netflix, and Hulu have reduced the effort needed by viewers to switch away from humdrum content. Yet, the ease of channel surfing began much earlier. In 1950, the Zenith corporation manufactured the first TV remote control named “Lazy Bones” that enabled channel changes without getting up to turn a dial (Zenith, n.d.). The desire for, and ease of finding, interesting content appears to have accelerated since then. While the data are not conclusive, the heavy use of the internet may be shortening attention spans as people search for more compelling content (Paul et al., 2012; Bradbury, 2016). Some reports show that 50%–60% of viewers turn off YouTube videos by their half-way points (Lang, n.d.).

The streaming platform Quibi, launched in 2020, hosted content that was only 10 min or less. Founded by media mogul Jeffrey Katzenberg, the company raised $1.75 billion from investors who believed short-form content would capture a generation accustomed to getting news and entertainment on demand. Nevertheless, Quibi was unable to sustain an audience, shutting down after 8 months (Williams, 2020). Was Quibi’s failure, and those of similar services, due to the length of content, the quality of the content, or the delivery platform (Chen et al., 2017)? One way to examine this issue is to identify why people stop watching content.

Most experiments that use videos to affect participants’ emotional states or prompt behaviors require that the entire stimulus be viewed (Barraza and Zak, 2009; Mar et al., 2011; Barraza et al., 2015). This approach, while convenient for the researchers, lacks validity since people do not always completely consume content. The present study gave participants the option to watch as much or as little of a set of videos and included an observable decision option after each video stimulus. By making the behavioral component optional, the study sought to relate how long participants viewed content to their likelihood of responding to the information shown in it.

Why people stop or continue consuming content is poorly measured by self-reports of “liking” or social media posts (Hollis, 1995; Galdi et al., 2008; Plassmann et al., 2015). The most prominent reasons to suspect inaccurate self-reports are the inability to accurately identify one’s affective states and that people often provide socially desirable answers to queries (Arnold and Feldman, 1981; Dang et al., 2020). An alternative approach is to measure neural responses during stimulus viewing. The present study measured neurophysiology in order to assess why participants spent time watching a video and to test if neural measures were associated with post-stimulus behavior. The unprecedented amount of available video content adds urgency to the development of an understanding of why people continue to watch video content when they have an option to stop. Indeed, videos shared on social media have been shown to affect others’ emotions via contagion showing their sustained impact (Kramer et al., 2014). Our approach seeks to discover a “keep watching” neurologic response in order to identify a mechanistic model that is more likely to generalize than self-reports.

Start/stop decisions appear to depend on a brain network that uses orientation to a stimulus (striatum), arousal (anterior cingulate), and executive function (prefrontal cortex; Konishi et al., 1998; Bush et al., 2000; Wunderlich et al., 2012; Cho et al., 2016; Gourley and Taylor, 2016). Indeed, 17% of those between 18 and 25 years old—those with immature prefrontal cortices—spent more than 20 h a week watching videos in 2020 (Statistica, 2022). The duration and frequency of video consumption have been associated with increased neural activity in orientation regions and decreased activity in a region associated with aversive stimuli (anterior insula; Tong et al., 2020). Stopping stimulus viewing can be conceptualized as a response to a specific neural signal, or as a degradation of the value associated with the stimulus, or perhaps both as this research is embryonic (Zandbelt and Vink, 2010; Sebastian et al., 2017, 2018).

Linking neurophysiologic responses to an observable post-stimulus behavior increases the likelihood that captured neural responses are signal and not noise (Cacioppo et al., 2000). For example, participants can be offered an opportunity to respond to a video or text stimulus by donating to a charity, investing in an African entrepreneur, or purchasing a product (Kraig et al., 2018, 2019; Falk and Scholz, 2018; Morris et al., 2019). While many studies of video influences on behavior have relied on self-reported engagement (Yu et al., 2018; Wohn and Freeman, 2020), this induces an endogeneity problem: participants who spend more time watching will report more engagement in the stimulus. Indeed, self-reported “engagement” or “interest” or “liking” are typically weak predictors of post-stimulus behavior (Li and Baker, 2018). At the same time, the “free to stop” approach in the present research induces a possible incentive problem: participants might choose to terminate video viewing in order to finish the experiment as quickly as possible. Thus, the approach we have taken biases the study against finding an effect.

### Model and predictions

In order to generate a testable hypothesis, we propose a simple mathematical model relating neurophysiologic responses to post-stimulus decisions. The model is a static version of the classic Hodgkin-Huxley model of the propagation of action potentials between neurons (Schwiening, 2012; Gerstner et al., 2014) that has been modified to capture the effects of neurophysiologic responses and time on choices. Let *p* be the probability of taking an action after a stimulus, *p* ∈ (0,1). Define *n* as the neural response to the stimulus *n ∈ ℝ*^+^, let scalar *d* > 0 be a neural threshold parameter, and let *t* ≥ 0 denote time. Then, the sigmoid function *σ*:ℝ*→*(0,1) maps the neural response and time for a given threshold d into a probability,


$$p=\sigma \left(n\left(t\right)\right),t;d)$$

**Figure 1:**
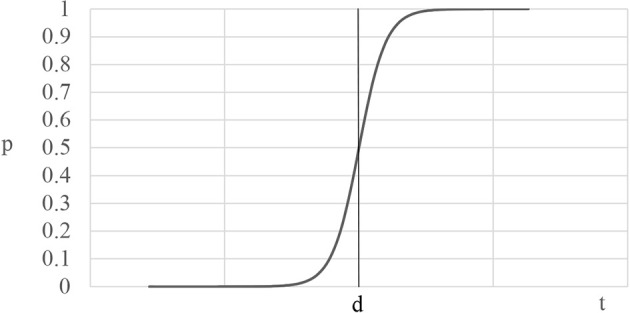
The curve shows the neural response (*n*(*t*)) during the time one views a video (*t*). When the Acceleration threshold (*d*) is passed, the probability of a post-video action rises rapidly.

The probability of taking an action increases with neural activation in response to the stimulus; that is, *p* is increasing in *n*(*t*) and *t*. A key prediction of the model is that the likelihood of deciding to act remains low when the neural response is small, but the cumulative effect of the neural response increases with time. The behavioral response is driven by the hysteresis that occurs at threshold *d* in which the probability rapidly changes from low to high. The model predicts that both time and neural responses increase the probability of taking an observable action and that the probability of an action rises after threshold *d* is passed. We will call threshold *d* “Acceleration” to make the analysis easier to read. The model guides the empirical approach that operationalizes Acceleration, described below. It also indicates that time will mediate the impact of neurophysiologic responses on behavior, further circumscribing the empirical analysis. Figure 1 illustrates the model relating neural responses and time into the likelihood of a decision.

## Methods

### Participants

Sixty-two participants (46% biological female) were recruited from the Claremont Colleges and surrounding community using our standing subject pool (age: *M* = 28.01, SD = 12.64). This study was approved by the Institutional Review Board of Claremont Graduate University (#3444) and all participants gave written informed consent prior to inclusion. The data were anonymized by assigning an alphanumeric code to each person. After consent, participants were seated at partitioned computer stations with headphones and were fitted with PPG (photoplethysmography) sensors (Rhythm+, Scosche Industries, Oxnard, CA). They then completed a short survey on demographics and mood using Qualtrics software (Qualtrics International Inc, Provo, UT).

Once all participants had completed the survey, they read instructions about the study and the decisions they would make. Participants were informed that they would watch a series of videos about social issues produced by nonprofit organizations. The instructions stated that they would earn $3 for each of the 11 videos that they watched. After each video started, they could decide when to stop watching it. At video termination, participants were prompted via software to decide if they wanted to donate some or all of the $3 they earned for watching the video to the charity that produced the video. The instructions emphasized that all donations were voluntary and would be sent to the featured charities at the end of the study.

**Figure 2:**
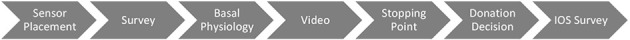
Timeline of the experiment.

After reading the instructions and being offered a chance to ask questions, participants closed their eyes for 3 min to measure basal neurophysiology. The videos started 30 s after a verbal instruction telling participants to open their eyes. At the end of the study, participants were paid in private $33 minus their donation amounts. There was no deception of any type and donations were made to featured charities at the study’s conclusion. Twelve participants were excluded due to missing neurophysiologic or decision data; nine of them in the same session due to a software glitch. The final dataset includes 50 participants. Figure 2 shows the timeline of the study.

### Neurophysiology

PPG data were sent via a Bluetooth hub to a commercial neurophysiology platform (Immersion Neuroscience, Henderson, NV). Neurologic Immersion combines signals associated with attention and emotional resonance to a stimulus that was identified in studies of neurochemical and electrical signals that predict social behaviors, including charitable donations (Zak et al., 2007; Barraza and Zak, 2009; Lin et al., 2013; Barraza et al., 2015; Zak, 2020) as well as mood (Merritt et al., 2022). The data were collected at 1 Hz. The Immersion Neuroscience platform uses variations in heart rhythms to infer neurophysiologic responses of the cranial nerves as a measure of the neural value of social experiences (Zak and Barraza, 2018).

### Instruments

A short demographic survey was followed by an assessment of dispositional empathy using the Interpersonal Reactivity Index (IRI; Davis, 1983) and basal affect using the Positive Affect Negative Affect Schedule (PANAS; Watson et al., 1988). After each video, participants completed the Inclusion of Others in Self (IOS) instrument that uses Venn diagrams that vary in their degree of overlap to capture the alignment of a participant with the issue shown in the video (Aron et al., 1992).

### Videos and data collection

The staff of a charitable donation hosting platform chose 11 videos from their site for use by the researchers. The topics varied from homelessness to food insecurity to awareness of polluted drinking water. Nine of the videos lasted from 120 to 444 s. Two videos ran for 13–15 min. These were edited by the researchers to run approximately 420 s in order to fit into the range of the other stimuli. PsychoPy toolbox for Matlab (Mathworks Inc., Natick, MA) was used to present the stimuli and to synchronize choice termination with physiologic data. The video order was counterbalanced.

### Statistical analysis

Participants had a free choice to stop watching videos at any time. Each of the nearly 700 time series for immersion was reviewed to ensure the time stamp for video termination aligned with the cessation of neurophysiologic data collection. Immersion during the video corresponds to the variable *n* in the mathematical model. The threshold *d* (Acceleration) in the theoretical model was operationalized using the cumulation of neurologic Immersion above a threshold value, similar to previous research (Merritt et al., 2022). That is,


$$Acceleration_{ij}=\int_{t=0}^{T}\left(n_{ijt}>M_{i}\right)d_{t}$$

where *n_ijt_* is neurophysiologic Immersion for participant *i* at time *t* while watching video *j* until time *T*, *M_i_* is the median of immersion plus 0.5 standard deviation for participant *i* across all videos. In simpler terms, Acceleration quantifies the highest immersion parts of each viewing experience by cumulating the peaks of Immersion above the threshold *M_i_* as in previous research (Merritt et al., 2022). The threshold for *M_i_* was determined by examining the correlation with time viewing videos. The results of the statistical analyses continue to hold for moderate changes in the definition of *M_i_*.

The data are an unbalanced panel due to differing video stop times. The analysis begins with tests of mean differences between donors and nondonors using Student’s *t*-tests (for readability, denoted “*t*-test”). Parametric relationships were examined using correlations and logistic regressions. A logistic regression with bootstrapped standard errors was estimated to establish the predictive accuracy of the mathematical model. Since the mathematical model predicts that time and neurophysiology both influence the donation decision, a mediation model was estimated to examine this relationship. Finally, sensitivity analyses were conducted by adding control variables age, sex, income, and positive and negative affect to determine if these factors influenced the time watching the video and/or the donation decision.

## Results

### Donations

The average donation across all 11 videos was $0.21 (SD = 0.62, *N* = 550). The average donation varied substantially across videos, from $0 to $0.46. On average, participants donated to 1.44 of the 11 charities shown in the videos (SD = 1.90). Whether a video elicited a donation also varied substantially, from videos with zero donations to 15 participants who donated money for the most effective video. There were no differences in the IRI subscales of personal distress (PD) or empathic concern (EC) between those who donated money compared to those who did not donate (Donators: PD: *M* = 8.90, SD = 2.96; EC: *M* = 16.90, SD = 3.43; Non-donators: PD: *M* = 8.53, SD = 2.22; EC: *M* = 16.26, SD = 4.27; PD: *p* = 0.64; EC: *p* = 0.57). Participants reported they felt closer to issues to which videos they donated (IOS Donate: *M* = 3.13, SD = 1.40; Non-donators: *M* = 2.24, SD = 1.28; *p* = 0.000). IOS was significantly correlated with donations (*r* = 0.23; *p* = 0.000).

### Demographics

The total amount donated across all videos increased with participants’ incomes (*r* = 0.45, *p* = 0.003), and age (*r* = 0.25, *p* = 0.085). Biological sex and education did not affect donations (Sex: *p* = 0.254; Education: *r* = 0.14, *p* = 0.324).

### Time

**Figure 3:**
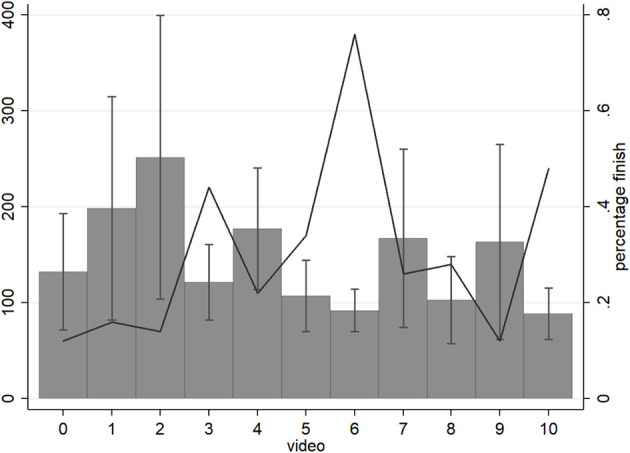
The time participants spent watching videos varied substantially (bars are SDs). The line shows the proportion of people who watched the entire video.

**Figure 4:**
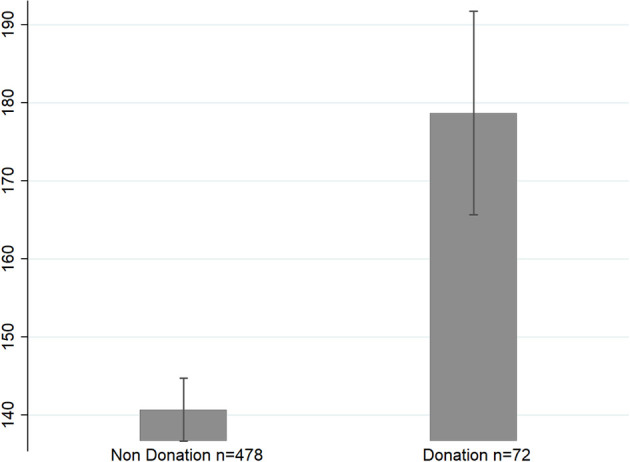
Videos that received donations were watched 27.0% longer on average than videos that failed to elicit any donations (*p* = 0.001). Bars are pooled SEs.

The free choice of time spent watching each video showed substantial variation, from 9 s to 444 s (*M* = 146.0, SD = 92.0) as did the proportion of participants who watched videos in their entirety (Figure 3). As predicted by the model, videos that received donations had significantly longer viewing times than videos that failed to generate donations (Donations: *M* = 179, SD = 111; No Donations: *M* = 141, SD = 88; *p* = 0.001). Indeed, the data revealed positive correlations between time watching a video and whether a donation was made (*r* = 0.14, *p* = 0.001; Figure 4) as well as time and the amount donated (*r* = 0.12, *p* = 0.005).

### Neurophysiology

**Figure 5:**
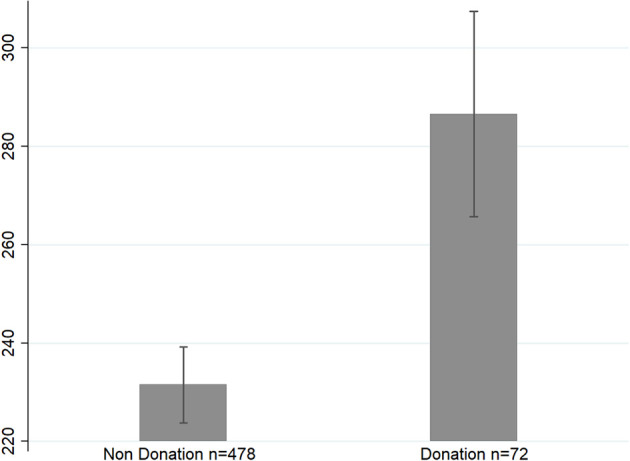
Average Acceleration during videos was 23.8% higher for those who donated money compared to those who did not donate (*p* = 0.011). Bars are SEs.

Average neurologic immersion while watching videos was unrelated to the donation decision (Donation: *M* = 4.05, SD = 0.682; No Donation: *M* = 4.03, SD = 0.650; *p* = 0.785). However, average Acceleration was higher when donations were made (Donation: *M* = 287, SD = 177.1; No Donation: *M* = 231, SD = 170.2; *p* = 0.011; Figure 5). Acceleration and Immersion were positively correlated (*r* = 0.412, *p* = 0.000) with the time watching videos positively related to Acceleration (*r* = 0.73, *p* = 0.000) while Immersion was not (*r* = 0.04, *p* = 0.400).

### Mediating effects of time

**Figure 6:**
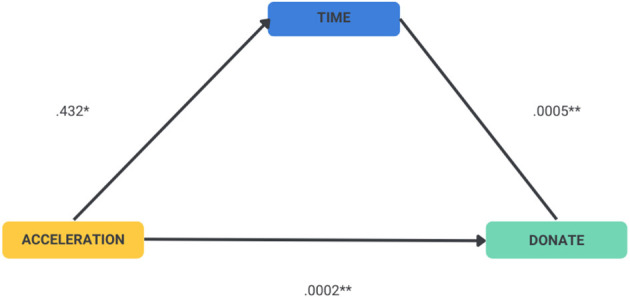
Estimation of the direct effect of Acceleration on donations and the mediating effect of Acceleration on time to watch videos. *p < 0.05, **p < 0.01.

The effect of time was examined by estimating a mediation model (Agler and De Boeck, 2017). We focused on Acceleration rather than on Immersion as the former was associated with donations while the latter was not. The mediation model showed that Acceleration directly influenced the time spent watching videos. In addition, Acceleration mediated the donation decision by increasing the time spent watching (Figure 6, Table A1). The results continued to hold when covariates IOS, age, income, and sex were included in the analysis (Table A2).

### Predicting donations

We estimated a logistic regression to assess the individual impacts of Acceleration and time on donation decisions. Acceleration was significantly associated with donations (*β* = 0.0017, SE = 0.0006, *N* = 550, *z* = 2.71, *p* = 0.007; Pseudo *R*^2^ = 0.014). The estimation produced a log-odds ratio of 1.0017. That is, a one-unit increase in Acceleration while watching a video was associated with a 0.17% increase in the likelihood of a donation. The Acceleration continued to be positive and significant (*β* = 0.0025, SE = 0.0009, *N* = 473, *z* = 2.81, *p* = 0.005; Pseudo *R*^2^ = 0.12) and has a higher log-odds ratio (1.0025) when including as controls an indicator for video finishing (VF), age, income, sex, and IOS (VF: *β* = −1.218, *p* = 0.003; IOS: *β* = 0.234, *p* = 0.018; age: *β* = 0.266, *p* = 0.011; income: *β* = 0.121, *p* = 0.066; sex: *β* = 0.321, *p* = 0.286).

Time also had a positive effect on the donation decision in a logit estimation (*β* = 0.0039, SE = 0.0012) with a similar log-odds ratio (1.004). The time watching a video continues to be positive and significant (*β* = 0.0051, SE = 0.0014) when controls are included (VF: *β* = −1.168, *p* = 0.002; IOS: *β* = 0.226, *p* = 0.023; age: *β* = 0.253 *p* = 0.015; income: *β* = 0.128, *p* = 0.051; sex: *β* = 0.354, *p* = 0.240), with an increased log-odds ratio (1.005).

## Discussion

The present study sought to add ecological validity to studies of the behavioral influence of messages by permitting participants to stop watching content at anytime without penalty. Indeed, busy, bored, or impatient participants would be expected to watch only a few seconds of each video to quickly earn money. Yet, nearly every participant watched video number six completely, which focused on homelessness, and nearly 50% of participants completely viewed two other videos. Consistent with previous studies of donations after messages about social ills, the videos prompted some participants to donate the money they had earned, though less than when entire videos were required to be watched. For example, in a study in which participants watched 15 videos completely and earned $3 for each, average donations were $0.54 in the control condition compared to a $0.21 average donation in the present research (Lin et al., 2013).

Our key finding was that the decision to continue watching a video depended on the neurologic value of the experience. Value was measured by a threshold variable we created, the Acceleration of Immersion. This approach is an extension of earlier work by our group that found the peak amount of Immersion, rather than average Immersion, was more predictive of people’s mood and energy (Merritt et al., 2022). The present analysis demonstrated that participants spent longer watching videos when Acceleration was high. As immersion appears to capture the neural value of social experiences (Zak, 2022), videos were watched longer when they generated Immersion peaks in participants. Furthermore, videos that had high Acceleration were those most likely to receive donations as shown in the mediation estimation. That people choose to extend the time they spent consuming content that was valuable to them is unsurprising. Our contribution is to show how neural value can be measured using a commercial platform and that increased value leads to an observable costly behavior.

An articulate argument for time as a proxy for value has been made by experienced design theorists supporting our findings that value mediates time watching videos (Pine and Gilmore, 1999, 2019). Valuable experiences typically have unexpected or surprising elements (Poulsson and Kale, 2004). These aspects of valuable experiences were likely to have been captured by the Acceleration variable we created. The brain responds to contrasts (Cacioppo and Cacioppo, 2020) and Acceleration may capture the point at which the brain can differentiate a mediocre experience from a valuable one.

Experiences that are relevant to individuals tend to be more valuable to them (Poulsson and Kale, 2004). The neural value of a video was affected by closeness to the featured issue as evidenced by the positive and significant correlation between acceleration and IOS (0.41, *p* = 0.000). This reveals another factor that affected how long participants watched a video. The Acceleration-closeness relationship may reflect “top-down” control in which additional neural resources are devoted to processing relevant information. For example, a functional brain imaging study showing photographs of objects documented greater neural activation when smokers viewed a picture of a cigarette pack compared to nonsmokers’ responses (Engelmann et al., 2012). Closeness to an issue or presenter also increases charitable donations after a request (Winterich et al., 2009; Baek et al., 2022). Our findings support the view that relevance influences how long people watch videos and how much they are influenced by them.

Curiously, the average time watching videos increased with participant age (*r* = 0.094, *p* = 0.03). The median age of participants was 25 while only 7% of sample was over 60 years old so this effect may be an artifact of the study population. Yet, there was no relationship between Acceleration and age (*r* = 0.06, *p* = 16) indicating that our findings were not driven by the behavior of the oldest participants.

Consistent with previous research, those who were older and had higher incomes donated more to the featured charities. That age affects donations is well established, while those with higher incomes tend to donate more to charity in absolute terms (Shelley and Polonsky, 2002; McClelland and Brooks, 2004; Snipes and Oswald, 2010; Zak et al., 2022). We did not find that participant’s biological sex affected donations, counter to much of the published research reporting that females donate more than males (Piper and Schnepf, 2008) while other studies find no effect (Barraza et al., 2015). Our finding may be due to the option to stop watching videos as there is some evidence that men are more impatient than women, though there is substantial variation and findings are quite context- and measurement-dependent (Silverman, 2003; Dittrich and Leipold, 2014).

There are several limitations of the present study, including the modest number of stimuli used, the use of videos focused on social issues, and the relatively homogeneous set of participants. Future research should examine the neural mechanisms that induce people to watch videos with a free choice to stop using other demographic groups and different video stimuli to confirm our results generalize. Nevertheless, the methodological approach described herein is a useful way to understand why people watch or listen to entertainment content and continue doing so.

There are several implications of our research for content creators. For example, our approach could be used to measure and edit content in order to increase neurologic Acceleration in order to keep people watching. Increasing the time spent watching videos is likely to increase the value of advertising during them and may more effectively influence consumers to take actions such as purchasing advertised goods or services. Advertisers could also use the methodology herein to build brand attachments which have been shown to increase how much people are willing to pay for products (Barraza et al., 2021). In addition, schools are increasingly using videos to transmit information, and watching a complete video rather than stopping it can increase student comprehension (Steffes and Duverger, 2012). Consumers want to have extraordinary experiences, whether in-person or online (Zak, 2022), and identifying objective measures of what people value is an important first step in creating such experiences.

## Data Availability Statement

The data are available at Open ICPSR https://doi.org/10.3886/E180661V1.

## Ethics Statement

The studies involving human participants were reviewed and approved by the Institutional Review Board of Claremont Graduate University (#3444) and all participants gave written informed consent prior to inclusion.

## Author Contributions

PZ: conceptualization, methodology, and software. RN: data collection. L-HL and RN: formal analysis. L-HL and PZ: writing—original draft, writing—review and editing. All authors contributed to the article and approved the submitted version.

## Figures and Tables

**Table A1:** A mediation estimation supports the neurologic model.

	**(1)**	**(2)**	**(3)**
**Variables**	**Donation**	**Time**	**Donation**
Acceleration	0.000212**	0.432***	
	(8.77e-05)	(0.0167)	
Time			0.000512***
			(0.000181)
Constant	0.0802***	42.59***	0.0563**
	(0.0225)	(3.475)	(0.0269)
			
Observations	550	550	550
R-squared	0.012	0.652	0.019

A mediation estimation supports the neurologic model. Acceleration motivated participants to watch the video. The R-squared in stage 2 shows that most of the variation from the Acceleration indicator was absorbed by the video-watching time, and stage 3 verified that mediation effect. Robust standard errors are in parentheses and ***p < 0.01, **p < 0.05.

**Table A2:** The mediation model is re-estimated using control variables and is shown to confirm the previous result.

	**(1)**	**(2)**	**(3)**
**Variables**	**Donation**	**Time**	**Donation**
Acceleration	0.000292***	0.423***	
	(0.000106)	(0.0184)	
Time			0.000649***
			(0.000206)
Video finish	−0.124***	2.524	−0.124***
	(0.0324)	(5.827)	(0.0308)
IOS	0.0305**	2.865	0.0287**
	(0.0125)	(2.000)	(0.0125)
Age	0.0403**	3.412	0.0382**
	(0.0168)	(2.810)	(0.0166)
Income	0.0157**	-0.922	0.0164**
	(0.00771)	(1.233)	(0.00771)
Gender	0.0365	−1.915	0.0377
	(0.0312)	(5.225)	(0.0309)
Constant	−0.129***	32.97***	−0.148***
	(0.0497)	(7.806)	(0.0498)
			
Observations	473	473	473
R-squared	0.101	0.653	0.109

As above, robust standard errors are in parentheses and ***p < 0.01, **p < 0.05.
